# Data collection costs in industrial environments for three occupational posture exposure assessment methods

**DOI:** 10.1186/1471-2288-12-89

**Published:** 2012-06-27

**Authors:** Catherine Trask, Svend Erik Mathiassen, Jens Wahlström, Marina Heiden, Mahmoud Rezagholi

**Affiliations:** 1 Centre for Musculoskeletal Research, Department of Occupational and Public Health Sciences, University of Gävle, SE – 801 76, Gävle, Sweden; 2 Centre for Health and Safety in Agriculture, College of Medicine, University of Saskatchewan, 103 Hospital Drive, Saskatoon, Saskatchewan, Canada, S7N 0W8; 3 Department of Public Health & Clinical Medicine, Occupational and Environmental Medicine, Umeå University, SE-901 85, Umeå, Sweden

**Keywords:** Ergonomics, Posture, Inclinometer, Observation, Questionnaire

## Abstract

**Background:**

Documentation of posture measurement costs is rare and cost models that do exist are generally naïve. This paper provides a comprehensive cost model for biomechanical exposure assessment in occupational studies, documents the monetary costs of three exposure assessment methods for different stakeholders in data collection, and uses simulations to evaluate the relative importance of cost components.

**Methods:**

Trunk and shoulder posture variables were assessed for 27 aircraft baggage handlers for 3 full shifts each using three methods typical to ergonomic studies: self-report via questionnaire, observation via video film, and full-shift inclinometer registration. The cost model accounted for expenses related to meetings to plan the study, administration, recruitment, equipment, training of data collectors, travel, and onsite data collection. Sensitivity analyses were conducted using simulated study parameters and cost components to investigate the impact on total study cost.

**Results:**

Inclinometry was the most expensive method (with a total study cost of € 66,657), followed by observation (€ 55,369) and then self report (€ 36,865). The majority of costs (90%) were borne by researchers. Study design parameters such as sample size, measurement scheduling and spacing, concurrent measurements, location and travel, and equipment acquisition were shown to have wide-ranging impacts on costs.

**Conclusions:**

This study provided a general cost modeling approach that can facilitate decision making and planning of data collection in future studies, as well as investigation into cost efficiency and cost efficient study design. Empirical cost data from a large field study demonstrated the usefulness of the proposed models.

## Background

There is widespread acceptance that in occupational studies, not all biomechanical exposure assessment methodologies are equal; most readers will be familiar with the ‘validity hierarchy’, which lists direct measures at the top, followed by observation methods and then self-report [1-6]. This hierarchy generally forms the basis of validation studies [7,8] and studies modeling the determinants of exposure [9,10]. However, validity is not the only criterion when selecting an exposure assessment method. The cited studies also acknowledge that the cost of some methods prohibit measurements over large numbers of people, for example, in epidemiological studies. Despite the ubiquitous challenge of budgeting for successful research, issues of exposure assessment cost are not often represented in the literature.

A recent review attempted to summarize the state of inquiry into cost-efficient exposure assessment of occupational exposures and identified only nine articles systematically addressing this area [11,12]. Since literature was sparse, the authors described the related but distinct notion of statistical efficiency, where researchers may use variance components to allocate measures in the most efficient way, based only on criteria related to statistical performance. The reviewers point out that such studies often make a series of – at times implicit – assumptions that are not borne out in reality: assuming that all measurements have an equal cost [13], or that costs can be assessed with only a few types of costs such as ‘measurement’ and ‘recruitment’ [14-17]. These works represent a substantial contribution to the area of cost-efficiency in research planning, but it is expected that the cost models may still be too simplistic [11]. To our knowledge, only one study has appeared, which employs a comprehensive cost model (i.e., including several types of costs) developed with empirical cost data to compare the cost-efficiency of four different techniques for observing postures from video [12]. That study clearly demonstrated that a simplistic cost model lead to other conclusions than a more complete model. So far, no study used comprehensive assessments in a comparison of basic exposure measurement methodologies.

Much of the cost-efficiency research to date has been performed with arbitrary measurement costs to demonstrate the principles. However, in order for researchers to make decisions about which method to use, realistic cost information is required. Despite its importance, research costs are not often discussed or reported in studies of occupational exposure. In their review, Rezagholi and Mathiassen [11] identified only two articles that explicitly listed the cost of measurement [14,18], and only one that did so for biomechanical exposures [18]. After the publication of the review, even the cited cost efficiency comparison of observation techniques has presented empirical costs for collecting biomechanical data, *in casu* postures. Although rare in listing measurement costs for biomechanical measurements, the Trask et al. article also had many limitations [18]. For example, the cost estimates did not include the costs associated with recruitment, travel, or analysis, all of which represent significant contributors to total study cost. The format of the cost model also limited its applicability; rather than listing fixed and variable costs, the article listed only the average cost for all measurements in the study, thereby limiting the applicability of the cost model to situations with different numbers of subjects or repeated measurements in identical settings. Some, but not all, of these limitations were resolved in the recent study of observation methods by Rezagholi et al. [11]. Ideally, a cost model will demonstrate how measurement allocation affects the total study cost as well as the tradeoff between cost components. A complete cost model with realistic cost inputs that can be applied to multiple scenarios would be a helpful contribution to this area of research.

One of the overarching goals of our research on cost-efficient exposure assessment is to investigate the costs of exposure assessment in field-based studies of musculoskeletal risk factors. To this end, the current study develops a new generic model for estimating costs associated with biomechanical data collection and uses that model to investigate the costs associated with collecting data using three commonly-used methods of assessing daily postural exposure: self-report via questionnaire, observation via video film, and direct measurement using inclinometers. Also, the paper addresses the use of the cost model in simulating different study design scenarios, thus providing decision support for efficient study planning. The basis for the two latter objectives were an extensive data collection of trunk and shoulder posture in airport baggage handlers during which costs were tracked *a priori* for all research activities.

## Methods

### Modeling costs

The cost analysis presented here focuses on the data collection stage of research. For the purposes of this cost analysis, the ‘project’ starts with the acquisition of equipment and pilot testing and ends with the completion of all scheduled measurement days. Activities such as preliminary meetings with industry stakeholders and writing grant proposals are not included in this analysis, nor is the processing of collected data into exposure metrics, the analysis of video, statistical analyses, or the reporting of results to the scientific community. Measurement costs were assessed for capital including all equipment, as well as all labour involved in several different tasks directly related to data collection: measurement planning, training, piloting, subject recruitment, travel, and the actual acquisition of data.

The cost model used in this study was developed using the economic principles employed in theoretical cost models previously published in exposure assessment science, and in particular the review on this topic completed by two of the authors of the present paper [11]. This theoretical framework was developed and refined by the author group based on previous experience tracking research time and costs during occupational exposure assessment [18,19].

The general model for assessing total cost for method *m*, C_T*m*_, included in the present case several fixed costs (denoted by Č): the cost of project meetings to plan the study (Č_M_); the cost of administering the research aspects of the study, including documentation, budgeting, and internal correspondence (Č_A_) and administration related to recruitment, including corresponding with employer and scheduling (Č_R_ ); the capital cost for equipment and software (Č_E_); and the cost of training data collectors to use measurement method *m* (Č_T_). The model also included several variable costs (denoted by Ċ): the cost of traveling to the worksite (Ċ_V_) and hotel accommodations during overnight trips (Ċ_H_); the cost of recruiting workers at the worksite (Ċ_R_); and the cost of onsite data acquisition (Ċ_D_). This model can be applied to any measurement method *m* following the general form of Eq. (1):

(1) $${\text{C}}_{\text{T}m}={\text{Č}}_{\text{M}}+{\text{Č}}_{\text{A}}+{\text{Č}}_{\text{R}}+{\text{Č}}_{\text{E}}+{\text{Č}}_{\text{T}}+\overset{}{{\text{Ċ}}_{\text{V}}}+{\text{Ċ}}_{\text{H}}+{\text{Ċ}}_{\text{R}}+{\text{Ċ}}_{\text{D}}\text{.}$$

Ċ_V_ is calculated as the product of the unit cost per trip ċ_V_ (which includes rail, taxi, and private auto costs to get to the worksite) and the number of trips n_t_; Ċ_H_ is calculated as the product of the unit cost per night ċ_H_ (including hotel and per-diem costs) and the number of nights n_n_; Ċ_R_ is calculated as the product of the unit cost of recruiting one worker ċ_R_ (including researcher time to locate randomly-selected workers at the worksite and invite them to participate) and the number of workers n_w_. There is a certain amount of preparation time that must be spent irrespective of how many workers are measured on a given day by one researcher. To account for this, Ċ_D_ is calculated as the sum of the costs for the first worker and concurrently measured workers as in Eq (2):

(2) $${\overset{}{\text{Ċ}}}_{\text{D}}{}_{=}\overset{}{{\text{ċ}}_{\text{S}}}{\text{n}}_{\text{m}}+\overset{}{{\text{ċ}}_{\text{F}}}{\text{n}}_{\text{d}}+\overset{}{{\text{ċ}}_{\text{C}}}{\text{n}}_{\text{c}}\text{.}$$

where ċ_S_ is the unit cost of measurement supplies (including tape for inclinometers, and questionnaire printing costs) per worker-day measured n_m_; ċ_F_ is the unit cost for the first worker measured on a researcher day n_d_; and ċ_C_ is the unit cost of concurrently measured workers on that day n_c_. The unit cost of the first worker measured includes confirming with the supervisor, setting up the research equipment, and waiting for the workers to arrive; this is consistent for each researcher-day no matter how many workers are measured on that day. The cost for additional concurrently measured workers includes the amount of additional time required to administer another questionnaire, video, or inclinometer measurement on the same day. If two researchers travel to the worksite on the same date, that is two researcher-days n_d_. Now, the full model can be expressed as Eq (3):

(3) $${\text{C}}_{\text{T}m}={\text{Č}}_{\text{M}}+{\text{Č}}_{\text{A}}+{\text{Č}}_{\text{R}}+{\text{Č}}_{\text{E}}+{\text{Č}}_{\text{T}}+\overset{}{{\text{ċ}}_{\text{V}}}{\text{n}}_{\text{t}}+\overset{}{{\text{ċ}}_{\text{H}}}{\text{n}}_{\text{n}}+\overset{}{{\text{ċ}}_{\text{R}}}{\text{n}}_{\text{w}}+\overset{}{{\text{ċ}}_{\text{S}}}{\text{n}}_{\text{m}}+{\overset{}{\text{ċ}}}_{\text{F}}{\text{n}}_{\text{d}}+\overset{}{{\text{ċ}}_{\text{C}}}{\text{n}}_{\text{c}}\text{.}$$

Because costs were collected for each measurement independently, estimates of cost variability (in terms of standard deviation) were available for ċ_V_, ċ_H_, ċ_R_, ċ_S_, ċ_F_, and ċ_C_.

### Applying the model to an example study: study population and data collection overview

Baggage handlers from a single employer at a large Swedish airport were recruited to the study. Eligible workers included full- or part-time workers, but not those on modified duties or a return-to-work schedule. Workers were stratified by workshift and invited to participate in the study in a randomized order. The worker population included both ‘ramp’ workers who loaded, unloaded, de-iced and maneuvered aircraft, as well as ‘sorting’ workers who packed luggage into cargo containers, unloaded luggage onto the arrivals belt, and drove baggage wagons. All subjects signed an informed consent form and all methods were approved by the Regional Ethical Review Board in Uppsala.

Twenty-seven workers were invited to have their trunk and shoulder posture assessed for 3 full shifts using three methods: self-report via questionnaire, observation via video film, and full-shift inclinometer registration via tri-axial accelerometers. Four trained researchers collected the data, including two of the authors (JW and CT) who supervised the data collection process. On each measurement day, a researcher planned to measure two workers, first mounting the inclinometers for full-shift measurement and then following each worker with a video camera for half a day. This strategy was selected to permit expansion of the sample size within the time span of the study and gather more exposure information in this population.

### Inclinometer data collection

Postural inclination with respect to gravity was measured using the VitaMove triaxial accelerometer system (2 M Engineering, Veldhoven, The Netherlands). A recorder was placed on the trunk between the shoulder blades and one on each upper arm over the medial deltoid. Workers came in before their shift for instrumentation and calibration, then were measured during their regular work tasks for the duration of the shift. Data were downloaded to a computer and backed up on a hard drive at the end of the shift.

### Observation data collection

Researchers video-filmed the workers continuously during one half of the shift. This involved following the workers while they performed their regular work tasks and endeavouring to capture their trunk and shoulder postures.

### Questionnaires data collection

Prior to starting their work shift, workers filled out a short questionnaire regarding their current perceived fatigue and body pain. After the work shift was completed, the workers filled out a post-shift questionnaire which repeated the fatigue and body pain questions, as well as including items on the amount of time spent performing specific postures and tasks during that particular shift.

### Cost data collection

The data collection of this study was designed for prospective cost tracking rather than retrospective accounting. This meant that our cost assessment could be based on empirical data rather than hypothetical amounts [20] and averages [18] as used in previous cost studies. Researchers tracked their own office and lab tasks such as administration, planning, training, and piloting were tracked using a custom Excel macro with defined task categories, which was subsequently compiled by one of the authors (CT) into hours per task category for all researcher staff working on the project. Researchers’ time spent travelling, as well as recruitment or measurement tasks was tracked via paper forms, and summarized for each researcher-day. Researchers were trained and coached in this process at the outset of the study, and a list of activities, definitions, and categories was jointly developed at the outset of the study. Cost tracking was reviewed by one of the authors (CT) throughout the data collection process and any questions or irregularities were discussed during team meetings. Although the reliability of researcher time tracking was not strictly recorded, there was no significant difference in task time reported between researchers. All costs were standardized to Euro currency using the average exchange rate between October 2010 to March 2011. Researcher time was valued at €31 per hour with a University overhead addition of 68%.

When determining the cost components for each method, data acquisition time was considered for each method independently. In practice several methods were applied concurrently, but the cost components presented assume that researcher time is paid for the full time spent on each method without savings for multi-tasking. This was designed to facilitate decision making about single-method studies.

The study also considered which stakeholders bore the costs of research. Although most of the costs listed were borne by the researchers, the employer paid for workers to come early and stay late on each worker-day (a total of 1 hour paid, non-productive time per day). Employer administrators also assisted with the study and tracked their time spent on research tasks using a customized MS Excel spreadsheet similar to that used by the researchers.

### Simulations based on study costs

The cost model can also be used as a decision tool to compare costs of studies with different sampling strategies. To demonstrate the effect of different study parameters on the total cost for each method and investigate the sensitivity of the model to different inputs, two kinds of simulations were performed: 1) changing the study design parameters (i.e. number of days in the field or measurement trips), and 2) changing the unit costs (the cost per trip or per first worker). In all cases, the simulation scenarios maintain the same number of workers and total volume of measurement data as the present data. These simulations illustrate the potential for cost savings with different study designs as well as giving examples of how researchers can use the model to estimate study costs given different situations. They also illustrate the sensitivity of cost estimations to changes in study conditions, as expressed in changed unit costs.

Table 1 details the parameters for simulations performed with the cost model. Simulation 1 examines the effect of traveling to and from the worksite for each of the 80 measurements (i.e. no accommodation at the worksite. Simulation 2 outlines the effect of measuring 1 worker per day on 80 researcher-days to reach 80 measurements. Simulation 3 looks at the effect of measuring 4 workers concurrently on 20 researcher-days to reach 80 measurements. Simulations 4-6 investigate the effect of different unit costs while maintaining the same study parameters as the present study. Simulation 4 examines the scenario where cost of travel and accommodation is set to zero, presuming that the measurement site is within biking distance from the university; Simulation 5 shows the scenarios where the cost of recruitment is increased 10-fold, assuming that it takes far longer to locate and persuade workers. Simulation 6 demonstrates the situation where the cost of equipment is set to zero, assuming that required equipment items have been previously purchased (recruitment, piloting, and training are still required).

**Table 1:** Study design parameters and selected costs for 6 different simulated scenarios for use with the cost mode All costs are in Euros

**Simulation designs**	**n**_**t**_	**n**_**n**_	**n**_**w**_	**n**_**d**_	**n**_**m**_	**n**_**c**_	**ċ**_**V**_	**ċ**_**R**_	**Č**_**E**_
Simulation 1: no accommodation	51	0	27	51	80	29	273	22	8431*
Simulation 2: 1 worker per researcher-day	45	60	80	80	80	0	273	22	8431*
Simulation 3: 4 workers per researcher-day	14	15	27	20	80	60	273	22	8431*
Simulation 4: No travel costs	30	0	27	51	80	29	0	22	8431*
Simulation 5: No equipment costs	30	38	27	51	80	29	273	22	0
Simulation 6: Recruitment costs set to 10-fold	30	38	27	51	80	29	273	220	8431*

n_t_ Number of trips to the worksite.

n_n_ Number of nights spent at the worksite.

n_w_ Number of workers.

n_d_ Total number of researcher days.

n_m_ Number of worker-days.

n_c_ Number of concurrently measured workers.

ċ_V_ Travel cost.

ċ_R_ Recruitment unit cost.

Č_E_ Equipment fixed costs.

* Fixed equipment cost for inclinometer. Observation Č_E_ = 4496 Questionnaire Č_E_ = 2401.

Other costs were kept at the observed values in the realized study.

## Results

### Study sample

As a result of 8 distinct recruitment efforts, 50 workers were invited to participate and 29 agreed. Twenty-seven of these workers were successfully measured. Out of 81 planned measurements (3 per worker), 80 were successfully completed, while one worker went on disability leave for a back injury before the third measurement could be completed. Thus, a total of 80 full workshifts were collected with successful, concurrent assessments using all three methods. Although the questionnaire and video recording were completed successfully each time they were attempted (success rate of 100%), the inclinometer method had a success rate of 93%. All participants were male; work shifts varied from 6 to 12 hours in length.

### Study costs

Values for the fixed and unit cost components observed for each of the three measurement methods are presented in Table 2. These cost components include costs borne by all research partners combined. The first column in Table 2 (‘applicable to all methods’) shows the costs that are required no matter which method is used; the total for each method in columns 3-5 represents the total study cost for applying that method, including the ‘applicable to all methods’ costs that are in the first column. Table 3 takes the present study’s total costs for all methods combined and estimates how much of the total study cost was borne by each of the study partners.

**Table 2:** **Cost model components for three measurement methods in the current study**^**a**^**(sd)**

**Cost component**	**Applicable to all methods^b^**		**Inclinometer**	**Video observation**	**Daily questionnaire**
Č_M_ (Planning meetings)	€ 2340				
Č_A_ (Administration)	€ 2548				
Č_R_ (Recruitment administration)	€ 2216				
Č_E_ (Equipment)	€ 2401		€ 6030	€ 2095	
Č_T_ (Piloting and training)	€ 4916		€ 1777	€ 693	€ 559
	*Unit cost mean (sd)*	*Study design parameter*	*Unit cost mean (sd)*	*Unit cost mean (sd)*	*Unit cost mean (sd)*
ċV (Unit cost per trip to worksite)	€ 273 (€159)	n_t_ = 30	…	…	…
ċH (Unit cost per accommodation day)	€ 149 (€22)	n_n -_ = 38	…	…	…
ċR (Unit cost of worker recruitment)	€ 22 (€14)	n_w_ = 27	…	…	…
ċS (Unit cost of measurement supplies)	…	n_m_ = 80	€ 1	…	€ 4
ċF (Unit cost of first worker measured)	…	n_d_ = 51	€ 508	€ 342	€ 135
ċC (Unit cost of concurrently measured workers)	…	n_c_ = 29	€ 83 (€ 49)	€ 216 (€ 70)	€ 9 (€ 8)
**C**_**T*m***_**Total Study Cost**^**c**^	…		€ 65342	€ 55 714	€ 36 925

^a^researcher, employer, and worker production costs combined.

^b^costs that are required no matter which method is used.

^c^total study cost for applying that method, including the costs in the first column.

**Table 3:** Proportion of costs borne by different stakeholders

**Cost component**	**Researchers**	**Employers admin staff**	**Worker’s production time**
Č_M_ (Planning meetings)	€ 1170	€ 1170,	…
Č_A_ (Administration)	€ 1897	€ 754	…
Č_R_ (Recruitment administration)	€ 1748	€ 468	…
Č_E_ (Equipment)	€ 10 526	…	…
Č_T_ (Piloting and training)	€ 7686,	…	€ 260
Č_R_ Recruitment time	€ 1413	…	…
Ċ_V_ & Ċ_H_ (Measurement travel)	€ 7659	…	…
Ċ_D_ (Measurement preparation)	€ 6456	…	€ 4162
Ċ_D_ (Measurement time and supplies)	€ 25 539	…	…
**Totals (in Euros)**	**€ 64 094**	**€ 2393**	**€ 4422**

### Costs of simulated studies

The total study costs estimated under the different simulated scenarios explained in Table 1 are presented in Table 4.

**Table 4:** Total study cost using the present study unit costs and different simulated design parameters

**Simulation scenario**	**Inclinometer**	**Video observation**	**Daily questionnaire**
Simulation 1: No travel or accommodation costs	€ 65 413	€ 55 705	€ 37 236
Simulation 2: One worker per research day	€ 85 040	€ 66 661	€ 48 192
Simulation 3: Four workers per research day	€ 44 372	€ 43 933	€ 25 464
Simulation 4: No travel and accommodation costs	€ 51 217	€ 41 509	€ 23 040
Simulation 5: No equipment costs	€ 56 911	€ 51 138	€ 34 764
Simulation 6: Recruitment costs set to 10-fold	€ 70 688	€ 60 980	€ 42 511

## Discussion

### Comparing cost of different methods

This study provided empirical cost data for use in cost models that can facilitate decision making and planning of future studies, as well as investigations of cost efficiency. For the current study design, exposure questionnaires were the least costly way to produce posture data, followed by observation and then inclinometer.

It is important to note that method-specific measurement costs are not always the most costly aspect of a study; in the present study the fixed and variable costs that apply to all methods make up between 43% and 78% of the total study cost (Table 2). This provides a substantially different result from a previous report of cost in ergonomic exposure assessment that disregarded travel and recruitment components, resulting in interview costs roughly 10% that of inclinometry [18]. Clearly this has a substantial effect on research planning and also the relative trade-off between methods; choosing a self-report method does not necessarily mean that one can conduct 10 times as many exposure assessment for the same price; one must consider the overall logistics of the study.

### The effect of study design and cost assumption

The simulations in Table 4 demonstrate the budget impact of several different situations. The general form of the model is intended to be a tool for researchers during project planning, and the empirical costs are intended to provide a guideline for similar situations. Although the potential inputs to the model are infinite, here we will use the simulations in Table 4 to comment on research planning and budgeting issues.

Study design and planning involves a lot of logistics, with some cost-relevant aspects seldom reported in the research literature. Decisions around sample size, measurement scheduling and spacing, concurrent measurements, location and travel, and equipment acquisition can have wide-ranging impacts on costs. Sample size affects generalizability, study power, and confidence in research results, but is limited by budget constraints. The **ċ**_**R**_ unit cost of € 22 included in the model quantifies explicitly the cost increase when a new worker is recruited. Retention and attrition are acknowledged problems in public health research [21], and it can be increasingly difficult to recruit and maintain participation. One of the simulated unit cost assumptions (simulation 6) examined a case where recruitment costs 10 times as much as in our study, resulting in an 8-14% increase in total study costs. It may be that as the sample size approaches an exhaustive sample of the worksite, the cost of recruitment does not stay constant as in the cost model presented here; costs may go up if employers are resistant to additional recruitment, or they may go down if social facilitation encourages workers to participate (i.e. they see their colleagues participating and want to join). These types of non-linear relationships have been hypothesized [20], but the current study did not collect empirical data to be able to implement them.

Although the inclinometers were robust enough not to require replacement over the span of the study, some types of equipment may require replacement that would make the direct measurement cost even higher. The inclinometer had the highest fixed costs, and therefore the most opportunity to amortize the fixed equipment costs over many measurements. The variable costs are also high since the inclinometer measurements were made for the full day, but this is a conservative estimate of time required for the inclinometer as this method only requires hands-on researcher input for set-up and take-down and occasional troubleshooting during the shift. This means the inclinometer lends itself very well to multi-tasking or to concurrent measurements. In multi-tasking researchers could analyze previously-collected data, write reports, or do administrative work during the seven hours between inclinometer set-up and take-down. If researchers were to perform useful work during the inclinometer measurements, a day’s inclinometer measurement would include only the cost of supplies and preparation time (approximately €175); the total study cost for an inclinometer study with the parameters of the current study would be €48 359, a savings of €16 983.

Work context could have an effect on the estimated costs, particularly for video tasks. Baggage handling work is very dynamic and takes place over a large workplace, so researchers must actively track workers with a camera for the entire measurement. This strategy would be similar in industries like health care, construction, agriculture, and resource industries such as mining. However, office work and assembly line jobs in manufacturing where the tasks are carried out in a small area may be good candidates for passive video capture using a tripod or a surveillance camera, in which case concurrent video measure would be possible. This would increase n_c_, the number of concurrent workers, and decrease the total study cost. If it were possible to have perfectly overlapping measurements that required no extra time, the total study cost would decrease slightly to 88-99% of current study costs. Despite a cost savings of 12%, concurrent measurement using video seems impractical, if not impossible under the conditions of the current study. In concurrent measurements, several workers would be measured within the same workshift for the same amount of preparation, travel, and waiting time and this could yield savings. Simulation 3 examines concurrent measurement of 4 workers per day and is the cheapest of the simulated options, costing 68-79% of the current study costs. Conversely, measuring only one worker per day (Simulation 2) requires more time and travel costs, resulting in costs 19-30% higher than the current study. The feasibility of concurrent measurements will depend on the start times of the workers and how flexible employer and/or workers are to modifying work times to allow for set up and measurement of several workers. Based on our experience, we estimate that for one researcher, 4 workers could be measured simultaneously using the inclinometer, 10 or more using the questionnaire, and only 1 additional worker for the video camera (unless the amount of recording time was shortened).

Although not explicit in the cost model, it is possible for the spacing of measurements to affect cost as well as the more well-known effects on exposure values [19]. In the current study we chose consecutive shifts to avoid the travel costs and administrative hassle of scheduling workers on a rotating shift schedule, but the trade-off is autocorrelation in the data. We also elected to conduct the study over a 3-month period during the winter, which surely impacts the type of exposures encountered and is unlikely to have full generalizability to the other parts of the year. However, a year-long sampling campaign would have been cost-prohibitive, given the need to retain part-time staff for the full year or retrain new staff.

Travel and accommodation needs are likely to vary substantially between research studies depending on the location of the researchers and institution relative to the data collection site. The current study showed substantial variation in travel distance and cost, represented by a ċ_V_ coefficient of variation of 58%. The distances in the current study required considerable travel, and introduced a trade-off between the total number of trips and length of stay. Simulation 1 shows the effect of making a new trip to the measurement site for each measurement day, which increases the costs less than 1%. Conversely, if each researcher made only 1 trip to the measurement site and stayed in a hotel for the duration of the study, costs decrease to 88-92% of the current study. If unit costs for travel or accommodation were zero because the measurement site was adjacent to the university as in simulation 4, the costs would be only 61-78% of the current study costs. Naturally cost is not the only factor when planning travel logistics and accommodation schedules; an additional consideration will be the tolerance of data collectors and local labour laws.

This study assumed no depreciation of the equipment cost, so the whole cost of research equipment purchase price is included in Č_E_. The costs presented therefore represent the assumption of ‘starting from scratch’ and having to purchase all equipment. However, were the researchers to pursue a similar study in the future, the decision between which method to select would be weighed without the fixed costs of equipment, since the equipment has already been acquired. This is demonstrated in simulation 5, where total study costs are only 87% of the current study cost for the equipment-intensive inclinometer method, 92% for the observation and 93% for the questionnaire.

### Who’s paying? Researcher- and employer-borne study costs

Over 90% of costs tracked in the current study were borne by researchers. It seems likely that time tracking biases would tend to overestimate this proportion, since researchers may be more motivated and diligent in reporting time spent on the study. It is possible that the time tracked by employer administrators (3.4% of the total study costs) is underestimated. For example, short tasks might be deemed ‘not worth tracking’ but cumulatively might represent a relevant cost. When industry stakeholders decide whether to participate in research, information about the time and resource commitments can help manage expectations and plan resources, as well as demonstrating researchers’ sensitivity to balancing business interests with research needs, the prioritization of which is different between researchers and industry stakeholders. Researchers forecasting costs for industry stakeholders may not have a strong influence on participant recruitment, but it seems likely to foster better trust and stronger relations between research stakeholders and almost certainly enhances retention or re-contact of study participants.

In the current study, employer administration and worker’s production time were both borne by the employer. In other contexts, the employer might not be able to pay for workers’ time, and transferring this cost (in terms of time spent or opportunity cost) onto workers is likely to affect the participation rate. The opportunity cost is the value of what is given up in order to participate in a study. Adding an hour to the workday may affect carpooling arrangements or transit/parking habits that can change direct costs of participation; it also means less time with family or sports and leisure pursuits. To address this, some studies provide an economic incentive equivalent to wage replacement [22]. This could increase the research budget substantially as worker production time accounted for over 6% of the total study cost in the current study. Although workers were paid for their time when they showed up early and stayed late, a limitation of the current study’s cost tracking method is that it does not account for opportunity costs associated with extending the workday or filling out questionnaires during work time. These types of costs are difficult to quantify and so were not included in the current study, although they may have an impact in participants’ decision making.

### Method performance: another consideration for decision making

If cost were the only consideration, the findings presented here would suggest that self-report is always the best option for assessing work postures. However, cost is far from the only criterion for selecting an exposure assessment method. It is possible for less tangible characteristics to render a method more desirable to worker, employer, union, insurance board, or regulatory stakeholders. These partners may have a *perception* that self-report will always be biased, that direct measurements are infallible, or that ‘expert’ observation is adequate in every situation. In order to foster collaboration, these perceptions need to be discovered and addressed either through education or compromise.

Researchers also need to consider the scientific quality of the data in terms of accuracy, precision, or predictive validity for health outcomes. In order to address this issue, the cost efficiency of each method must be determined by comparing the price to the performance of each method. To determine cost efficiency, cost data (such as that contained in this report) could be combined with components of variance from collected exposure data to quantify the cost efficiency of each method and sampling schemes as described previously [11,20]. Variance components are often used to guide allocation of measurement efforts within and between individuals [19,23]. Most studies investigating this issue have considered only statistical efficiency, not measurement cost [11]. However, optimization based on both costs and statistical performance could yield a substantially different study design than what follows from optimizing only with respect to statistical performance [16,17,20]. For example, when within-worker variance is higher than between-worker variance and recruitment costs for engaging participants are high compared to costs for collecting more data from subjects already in the study, multiple measures on fewer workers may prove to be a more cost efficient sampling strategy than that suggested when only statistical performance is considered, i.e. distributing measurements among as many subjects as possible [14,20]. Bias is also an important consideration; self-report has been shown to, in general, overestimate physical exposures [24], particularly in workers with musculoskeletal symptoms [25]. Some observation methods may also be associated with bias when compared to results obtained by inclinometry [12]. This type of misclassification could have deleterious effects on an epidemiological study of health outcomes. Previous cost-efficiency studies have shown that for simple models of cost and statistical performance, cost efficiency can be explicitly optimized. However, the complexity of the cost model, including possible non-linearity, and the structure and degree of errors in some exposure variables may preclude a fully-optimized cost efficiency model and instead favour a numerical rather than analytical approach.

Thus, future research should investigate not only the price, but also the cost efficiency of the exposure assessment methods in terms of value outputs like precision and bias of the resulting information.

### Performance of the cost model

The accounting protocols used in this study allowed estimation of variability in unit costs, and variability tended to be high. The coefficient of variation (CV) in recruitment and travel were both over 50%, demonstrating that these costs are influenced by other factors than just the number workers recruited or number of trips. The cost for concurrently measured workers was also highly variable (CV = 32-88%), a result of the varying amount of time required to complete an additional measurement. Together, the variability of the unit costs gives some insight as to the stability of total study cost if the study were to be repeated, introducing the notion of confidence intervals around a projected study cost. The uncertainty of cost estimations, as well as the impact of such uncertainty on total costs, could be an avenue for future research.

The Rezagholi and Mathiassen review lists several simplifying assumptions in existing cost models: assuming that all measurements have an equal cost [13], or that costs can be split into two or three stages or levels (e.g. ‘worker’ level and ‘repeated measures’ level), or can be represented by limited components such as ‘measurement’ and ‘recruitment’ [14-17]. The current model has attempted to expand on previous models by including these missing aspects. However, with nine types of costs included, it is worth considering whether the cost model could be simplified without hampering its performance. For example, the cost of supplies was very low, with a unit cost of €0-4 and a supply cost of less than 1% of the total study cost. Similarly, recruitment accounted for less than 2% of the total study cost. These costs would have so little impact on decision making that they are probably not worth tracking. The most important cost components are those involving researcher time. Thus, another issue for further research would be the cost efficiency of the cost tracking *per se*; for instance whether a simpler and cheaper cost tracking procedure would free resources in a study that could then be used to collect more exposure data.

There are a number of other ‘hidden’ costs that were not explicitly separated out in the model. For example, energy and infrastructure costs were considered to be included in the University overhead which was incorporated into researcher work time. As a result, researchers looking to use the model cannot separate institutional overhead components. This limitation could be navigated by adjusting unit costs for researcher time to apply different overhead values.

The data presented do not currently include the costs related to post-processing and analyzing collected data into postural exposure variables. Rather, the present study stops accounting when all the acquired electronic files are downloaded onto University servers and the questionnaires are delivered to University file storage. These additional costs in a full exposure assessment include all tasks between data collection and statistical analysis, including data entry for paper questionnaires, data processing, visual inspection and quality control for inclinometer data, and observer time spent recording postures from video still frames. A recent study investigating the cost-efficiency of different observation sampling protocols suggests these costs can be substantial, but also that they depend significantly on the technique used for data processing [12]. There are many options for processing and analyzing data depending on the research questions; the present results may be viewed as a common starting point for a complete cost assessment of basic data collection up to the point of finalized exposure data, where the processing costs differ depending on the techniques used for retrieving exposure variables. Since processing and analysis costs could comprise a considerable portion of total research costs, the comparisons between exposure assessment methods could evolve as these components are included.

Some time was spent multitasking in the current study, and it is difficult to separate the time spent on each method. For example, inclinometer and video data could be downloaded while the questionnaire was being filled out. Although it would seem that the inclinometer took the most preparation time, it is not always possible to separate how much time was spent on what. For this reason, the time estimates for each method (and especially the questionnaire) could be overestimated. Conversely, when questionnaire methods are applied alone, the time it takes to identify a participating worker and introduce an instrument is non-trivial but was not explicitly accounted for in our data, so overestimates on the researcher time spent on the questionnaire are likely minimal.

## Conclusion

The current study addresses research gaps in the area of cost efficiency [11] and improves on previous studies of cost [18] by introducing a comprehensive model for estimating total costs including fixed and variable cost components, implementing prospective collection of cost data, and acknowledging who bears the costs. In terms of determining the cost of common posture assessment methods, findings show that posture assessment by inclinometry was more expensive than observation, which was more expensive than self-report; the majority of costs were borne by researchers. However, costs for data processing and analysis are non-trivial and may have substantial impact on the total cost of research including both data collection (as in the present study), and subsequent processing of data into end-point exposure variables of interest; the costs of the processing method also needs to be considered when selecting an exposure assessment method.

This study successfully developed a cost model with cost components for the three exposure assessment methods; this model is intended to be used as a research planning tool for future ergonomics field research. The division of the cost model into components means that researchers can use information about their own specific unit costs, e.g. for travel and researcher time, and their proposed sampling strategy to tailor costs for a novel study, supporting decisions that lead to a more efficient resource allocation in studies of occupational ergonomics.

## Competing interests

None to declare.

## Authors’ contributions

CT developed the specific research question at hand, contributed to sampling strategy and data collection protocols, supervised data collection, drafted the cost model, processed the results, and drafted the manuscript. SEM conceived of the original study of flight loaders, developed the sampling strategy, contributed to data collection protocols, and contributed significantly to the manuscript. JW contributed to sampling strategy and data collection protocols, collected worksite data, and contributed significantly to the manuscript. MH contributed to sampling strategy and data collection protocols, and contributed significantly to the manuscript. MR took part in designing the cost model, interpreting the results, and contributed significantly to the manuscript. All five authors read and approved the final manuscript.

## Pre-publication history

The pre-publication history for this paper can be accessed here:

http://www.biomedcentral.com/1471-2288/12/89/prepub
